# Effect of an integrated maternal health intervention on skilled provider’s care for maternal health in remote rural areas of Bangladesh: a pre and post study

**DOI:** 10.1186/s12884-015-0520-5

**Published:** 2015-04-28

**Authors:** Nafisa Lira Huq, Anisuddin Ahmed, Nafis al Haque, Moyazzam Hossaine, Jamal Uddin, Faisal Ahmed, MA Quaiyum

**Affiliations:** International Centre for Diarrheal Disease Research, Bangladesh (icddr,b). 68, Shaheed Tajuddin Ahmed Sarani, Mohakhali, Dhaka 1212 Bangladesh; Department of Microbiology, Child Health Research Foundation (CHRF), Dhaka Shishu (Children) Hospital, Dhaka, Bangladesh

**Keywords:** Bangladesh, Maternal health, Remote rural area, Skilled provider care

## Abstract

**Background:**

An integrated maternal health intervention in a rural sub district of Bangladesh focused on the training and deployment of a required number of Community Based Skilled Birth Attendants (CSBAs). The aim of the study was to assess the effect of the intervention on use of skilled provider care during pregnancy, delivery and after delivery.

**Methods:**

The effect compared the skilled providers care in low performing areas with high and medium performing areas through pre–and post–intervention surveys. The post-intervention survey was conducted two years after the completion of the intervention. Village clusters, with populations of approximately 3000, were randomly assigned to the surveys. Mothers who had delivered within the 6 months prior to the surveys, were recruited for the structured interviews. Logistic regression was conducted to compare the effect.

**Results:**

Majority of mothers in the low performing areas belonged to a poor economic quintile. The level of skilled attendance for 4+ Antenatal Care (ANC) and delivery increased sharply from baseline to endline survey in low performing areas (ANC: 1.6% to 15.3%, p < 0.0001; skilled birth attendant at delivery: 12.6% to 38.3%, p < 0.0001). Less than 1% of the women received Post Natal Care (PNC) from a skilled provider prior to the intervention, and this proportion increased to 20% at the end of the intervention. Adjusted odds showed that the intervention had an effect on the individual performing area on use of skilled provider care during ANC, delivery and PNC. The increase of 4+ ANC from skilled providers and skilled birth attendants during delivery in the low performing areas due to the integrated maternal health intervention was significant relative to the increase in the high performing areas [4+ ANC from skilled providers – OR: 3.8 (1.9–7.6); skilled birth attendants – OR: 2.8 (2.1–3.8)].

**Conclusion:**

An integrated maternal health care intervention focused on deployment of a required number of CSBAs increased the opportunity for underprivileged women to benefit from skilled providers care during their pregnancy. This integrated intervention might improve the maternal health in developing countries where home delivery with unskilled attendants is common.

## Background

Bangladesh is committed to achieve Millennium Development Goals (MDGs) 4 and 5 to reduce childhood mortality to 50 per 1,000 live births and maternal mortality to 143 per 100,000 live births respectively by 2015. Although there is an encouraging decline in maternal mortality ratio (MMR) from 332 – 194 since 2000 – 2010 [1], Bangladesh still has a high MMR and this remains a challenge for the country. Bangladesh has a wide range of health care services especially in rural areas, where different tiers of health services have been established. In the Upazila (Sub district), the primary health care service is provided by the Upazila Health Complex (UHC), which has in-patient, out-patient and basic laboratory facilities. At the union level (village community level) there are Family Welfare Centres (FWCs), Rural Dispensaries (RDs) and Community Clinics [2]. In the UHC, 8–10 MBBS doctors and other support staff have been allotted. A paramedic, a medical assistant and a Family Welfare Visitor (FWV) are available to provide services at the union level health facilities. These facilities provide free services for maternal health, family planning, communicable disease control, child health and basic curative care [3-6].

The skilled birth attendant (SBA) program was initiated by the Government of Bangladesh in 2003 to improve safe delivery at home. The piloting of SBA training program was supported by WHO, UNFPA and Obstetric and Gynecological Society of Bangladesh. Ninety fieldworkers were selected from six districts for training in 2003 [7]. The experiences of an evaluation of this pilot program were the basis for extending the SBA training program in all sub-districts of 19 different districts in Bangladesh. In 2007, the Bangladesh Government provided the training program for 3000 community SBAs known as CSBAs [8]. The CSBAs were trained in midwifery skills to enable them to provide the basic level of skilled delivery care at home. This training program was six- months in duration. To reinforce the skills of the attendants, a three- month additional course was approved by the Nursing Council in 2007. This extended program provided training on management of bleeding in pregnancy, childbirth and post-partum, and dealing with unexpected problems and difficult deliveries, medical disorders and other factors contributing to complications, including infections in mothers and newborns. The government of Bangladesh committed to train 13,500 SBAs by 2010 [7].

According to the Bangladesh Demographic and Health Survey (BDHS), 2011, the four or more antenatal visits (4+ ANC) have increased from 22% in 2007 to 26% in 2011. Although this is still low, the proportion of births delivered at health facilities has been increasing rapidly since 2004: from 12% in 2004, to 17% in 2007 and to 29% in 2011. In Bangladesh, one-third of births are attended by medically trained personnel [doctor, nurse, midwife, FWV, or CSBA]. The proportion of deliveries attended by medically trained providers has doubled from 16% in 2004 to 32% in 2011, but that was mainly due to an increase in institutional deliveries. Similarly, postnatal checkup from a medically trained provider within 2 days of delivery has increased from 20% in 2007 to 27% in 2011 for mothers and from 20% to 30% for infants over the same time [9]. However, a large proportion of women in rural Bangladesh still prefer home delivery by traditional birth attendants and this proportion is highest amongst the poorest women [9]. Different socio-economic indicators were positively associated with the utilization of health services by studies in rural Bangladesh [10,11]. The inequity in the use of skilled attendance at birth was substantial and varied from less than 5% to 30% between the lowest and highest socio-economic quintiles [12]. Assistance by medically trained personnel at childbirths was high in urban areas and the Southern parts of Bangladesh [9]. Access to skilled maternal and child health services in the rural areas of developing countries is limited by the distance to health care services and the lack of autonomy of the women in household decision-making processes [13,14]. Since the early 1980s, private health care services have been established in Bangladesh, but are more frequently located in urban areas rather than in the remote rural areas [15,16].

CSBAs could assist childbirth [17] and this would be highly desirable where the majority of women give birth at home. They can also minimize risk of maternal death by immediate referral to facilities which provide Comprehensive Emergency Obstetric Care (CEmOC). In Bangladesh, post partum hemorrhage (PPH) is the leading cause of maternal death, followed by eclampsia [18]. According to the Bangladesh Maternal Mortality survey, 2010, PPH and eclampsia accounted for 31% and 20% of maternal deaths respectively [1]. Some essential management of these major maternal health complications, e.g. caesarean sections, blood transfusion, maintenance dose of drugs etc. cannot be provided at the community level. Nonetheless, ANC is recognized globally, as one of the key components in the safe motherhood package [19], through which pregnancy related complications can be prevented and managed. Moreover, for women who are not able to reach a health facility in a timely manner, initial management services can be offered at the community level by skilled health care providers. Community based skilled providers can screen the high risk mothers during ANC visits and provide loading dose of Magnesium Sulphate (MgSO4) to prevent eclampsia and subsequent fits in eclampsia. It may also be possible to provide drugs for treating postpartum hemorrhage and sepsis at the community level during an emergency prior to hospital referral [20].

One of the important requirements for an efficient rural health service is the requisite proportion of skilled health care providers at the community level [21]. The health services in rural areas especially in the remote rural areas, often suffer from serious shortages of doctors, nurses and other health service providers [22]. Effectiveness of maternal health interventions on maternal mortality by increasing the coverage of health care providers at community level has been reviewed in several interventions [23]. If the coverage of health care providers is effective in promoting skilled attendants at delivery, it should address the geographical distribution of services and affordability of such services. The distance and cost to travel to the health facility would make women of the most remote areas vulnerable if they opt to deliver at the health facility. Onset of labor may occur during the journey to the health facility, and it may either complicate the labor or exacerbate related complications [24]. In many developing countries, skilled attendants such as midwives, nurses and doctors are made available at close proximity to rural women. They are trained in midwifery to manage normal (uncomplicated) pregnancies, childbirth and the immediate postnatal period and identify, manage or refer complications in the woman and newborn [25,19].

The government CSBA program is assigned to improve maternal health and other components of reproductive health. However, the government of Bangladesh lacks the resources to achieve the full coverage of CSBAs for the women of Bangladesh. The policy of the government is to place one CSBA for a population of 8,000-10,000. For substantial contribution in improving maternal health by CSBAs, the private sectors and NGOs have implemented parallel training programs to increase the coverage by private CSBAs. This initiative is using a similar training program as the one issued by the government [Anwar I, Islam N: Maternal, Neonatal and Child Health (MNCH) Mapping study 2011. A Report. 2011 (Unpublished)].

In this background, the International Centre of Diarrheal Disease Research, Bangladesh (icddr,b) in collaboration with the government of Bangladesh, undertook an initiative to strengthen the existing health system through implementing an integrated evidence-based maternal health intervention package. The aim was to increase the use of skilled attendants at delivery, and skilled care during ANC and PNC, thereby decreasing the rich/poor gap in the use of skilled care at a selected sub district. The pregnant women of the selected sub district in Bangladesh were assisted by a required number of both public and private CSBAs. In addition to the existing government CSBAs in the sub district, icddr,b in collaboration with a local NGO, trained the required number of private CSBAs and evenly distributed them at their respective villages. In this intervention the CSBAs were trained on necessary midwifery skills, equipped with clean delivery kits (CDKs) and linked to the referral health facilities. The objective of this study was to investigate the effectiveness of the intervention on three major components of childbirth- ANC, delivery and PNC from skilled providers among mothers who are based at the remote unions of the selected sub district.

## Methods

### Study settings

The study was conducted between 2010 and 2011, in Shahjadpur, a sub-district of the Sirajgonj district in Bangladesh. It has a population of around 583,350 and is about 180 km northwest of Dhaka, the capital of Bangladesh. The sub district has 13 unions (lowest administrative unit of a sub-district) and one municipality area. Gala, Kuijury, Sonatoni and Porjona are the most remote unions in Shahjadpur. Gala, Kuijury and Sonatoni have an estimated population of 25,000–40,000 each, and Porzona has an estimated population of 70,000. Gala, Kuijury and Porzona are approximately 10–15 km away from the UHC, which is the primary level health facility of the sub district. Sonatoni is 25 km away from the UHC and is predominantly surrounded by the river Jamuna. Only one rural dispensary is situated in this union to provide primary healthcare services to local rural communities and prior to the introduction of this intervention, there was no CSBA in this union. One CSBA covered Gala and Kuijury each before the intervention. One non-upgraded FWC is present in Kuijury and one upgraded FWC in Gala. The upgraded FWC has facilities for normal delivery. The largest remote union, Porzona, had three CSBAs before the intervention and two rural dispensaries.

The study aimed to provide one CSBA for a population of 8,000–10,000 as per the policy of the government of Bangladesh. Therefore to ensure full coverage, at least 60 CSBAs were needed in Shahjadpur sub-district. Prior to the intervention, 28 CSBAs from the government were available and therefore an additional 32 CSBAs were required. The criteria of the government for eligibility for CSBA training are 1) married women, 2) minimum education level to 10th grade, 3) over 35 years of age and 4) interested to work in the community as a CSBA. The selection of CSBAs was conducted through a local NGO. Final selection of trainees was determined by a selection committee at Upazilla level formed by the government. The CSBAs were trained through a standard curriculum approved by the government and the Obstetrical and Gynecological Society of Bangladesh. Six months of theoretical and practical training were provided in fully equipped selected training centres of the government (Family Welfare Visitors Training Institutes and District hospitals). The training includes antenatal, delivery and postnatal care from skilled providers, identification and immediate management of complications with referral. Immediate management includes active management of third stage of labor, use of misoprostol for prevention of PPH, injecting loading doses of magnesium sulphate to control the eclamptic fits before referral, cardiopulmonary resuscitation and essential home based newborn care for sick babies. The CSBAs were certified and registered by the Bangladesh nursing council. The required numbers of private CSBAs were selected and trained in proportion to the population size of each union and the number of existing CSBAs. The recruitment process was completed between February and April 2009. The 32 private CSBAs were divided into two batches for training. The 1^st^ batch received the training during May to November 2009 and the 2^nd^ batch received the training during June to December 2009. The 1^st^ batch was deployed in Shahjadpur in December 2009 and the 2^nd^ batch in January 2010.

A package of interventions was integrated, that was implemented through the CSBAs. The integrated package included the following evidence-based interventions:

- Counseling (maternal and neonatal danger signs; 4 ANC visits; use of birth kit, skilled attendant at delivery; planning for emergencies -transport, funding and blood donor availability; at least 3 post-partum visits, at home or in facility- one visit within 24 hours).
- Updated safe delivery kit with a standardized delivery mat to measure post-partum blood loss so that excessive hemorrhage can readily be identified and referred; soap for hand washing by the birth attendant; 2 pairs of gloves for clean delivery; an educational card to help families recognize danger signs of delivery complications; as well as a checklist for preparation for the delivery.
- Management of eclampsia by introduction of loading dose of magnesium sulphate by CSBAs followed by referral.
- Prevention of hypothermia (drying, wrapping and skin-to-skin contact, delayed bathing for at least three days).
- Umbilical care (cord should be kept dry and clean. Nothing should be applied to the cord).
- Early initiation and exclusive breastfeeding, including feeding of colostrum and avoidance of pre-lacteal feeds.
- Identification of sick newborn and referral: Referral suggested for convulsions, unconsciousness, breathing difficulty, the baby is hot or cold to the touch, weak or absent cry, lethargic or less-than-normal movement, inability to suckle, skin pustules or redness, umbilical redness extending to the skin, or yellow colouration of the body.
- Extra care for preterm and low birth weight baby.

icddr,b funded the training of NGO CSBAs and they were deployed under the Government-NGO (Public- Private) partnership. At all stages of the study there was a community mobilization process through forming Community Support Groups (CSGs) by including community members. Information about the skilled maternal health services was provided through the CSGs in order to inform and prepare the community for the intervention. During the CSG’s meeting, the respective CSBAs were introduced to the local population. In addition to meetings, the volunteer members of the CSGs identified pregnant mothers at their household (HH) level and oriented mothers on skilled providers care during pregnancy, delivery and after delivery. After listing of pregnant mothers, the volunteers visited the mothers once a month. CSG meetings were also held once a month in their respective communities. Upazila and union based orientations were held where the CSBAs were introduced to the local government authorities (Union chairman and ward members) as well as the local elites. icddr,b collected process data throughout the intervention and also attended the monthly meeting of the government health department at the sub district level to illustrate problems identified by the process documentation. The data shared mainly on quality of services provided and functional status of the facilities in these meetings. Supportive supervision was made jointly by this public private partnership (PPP). Through such visits and monthly progress updates, the government health authority accelerated improvements.

### Study design and methods

This study was a pre- and post-test intervention in design. We conducted a household baseline survey on mothers who had given birth within the 6-months prior to the interview. Similarly, after two years of implementation of the intervention, an end line survey was conducted with the mothers of similar criteria to that of the baseline survey. The same methods and questionnaire were used in both the surveys. The questionnaire included socio-economic characteristics of the mothers including possession of household assets, demographic characteristics, care-seeking patterns for the last pregnancy/delivery/postpartum, newborn care, and neonatal outcomes.

### Sampling and sample size

The sample size for this pre- and post-test intervention study was calculated based on the objectives of the study. During the 2-year interval time period, to test a reduction of neonatal mortality from 37 to 21 per 1000 live births (with 95.0% confidence and 80.0% power, a minimum of 1250 mothers who delivered live born babies were required to be observed at each point of the surveys. To cover mothers who delivered still births (assuming 30/1000 births), a total of 1290 mothers who delivered either a live or still birth were also needed. Further increasing the sample size by 5.0% to compensate for non-response, a total of 1360 mothers who had delivery outcome (after 28 weeks of gestation) were required. Considering design-effect of 2 for cluster sampling - a total of at least 2720 mothers, who delivered in the last 6 months, were required in each baseline and end line survey. Thus, we interviewed 3158 and 3431 mothers during the baseline and end line surveys respectively. This sample size provided sufficient power to test an increase in skilled attendance from 13.0% to 50.0% (30 is the minimum number required to test the difference in proportion with 80.0% power and 95.0% confidence interval at each survey).

The 13 unions and one municipality area of Shahjadpur were divided into clusters, where each cluster comprised of approximately a population of 3000. The Field Research Supervisors (FRSs) drew a sketch map of the villages by marking major and minor roads, rivers, health facilities, schools, the union council’s office and other important features. Boundary features such as rivers or neighboring villages were also marked. This generated 200 clusters out of an estimated total population of 583,350 in Shahjadpur sub-district. Out of the 200 clusters, 80 and 100 clusters were randomly selected for the baseline and end line surveys respectively.

A two-stage sampling design was adopted in the study. The primary sample unit was the cluster of a population of approximately 3000 mapped by the FRSs. The secondary sample unit was at the HH level. The FRSs developed a list of all mothers who delivered live/still births in the last 6-months by HH visit in selected clusters. Simple random sampling was used for selection of the required number of samples from the HH list. The FRSs interviewed the mothers who had given birth within the last 6-months at the randomly selected HH level.

### Data collection

The baseline survey was conducted between January to March 2009 and the end line survey was conducted between March to June 2012 using a structured questionnaire. Questions validated in other maternal health surveys were included in the questionnaire. After recruitment of 16 Field Research Assistants (FRAs), the research team trained the FRAs as well as the FRSs. The training consisted of lectures on the structured questionnaire, interviewer skills, methods of data collection, negotiation skills and team building. The questionnaire was pre tested in selected villages which were not part of the study by the FRAs and also by the FRSs. The research team and the FRSs closely monitored and supervised the FRAs at HH level during data collection and checked the consistency of the responses of the questionnaire.

### Measures

#### Performing areas

Based on the use of skilled provider attendance at delivery (facility delivery and skilled attendance at home delivery), the whole Shahjadpur sub district was divided into three different performing areas—high, medium and low. Other indicators for the use of skilled providers in ANC and PNC were also highest in the high-performing areas and lowest in the low performing areas. This division was based on the results that emerged in the baseline survey. The high-performing area consists of 4 unions, including the municipality. Most of the health facility services are concentrated in this area. The low-performing area comprises 4 unions in the south-eastern part of the sub district which are heavily affected by erosion of the river Jamuna and are hard to reach. The rest of the unions in the north and south western part of the sub district were categorized as a medium-performing area on the basis of the selected maternal health indicators.

#### Outcome variables

Three indicators on the use of maternal health services were used in this analysis: use of antenatal care during pregnancy by a skilled provider, skilled attendance at delivery (this included institutional deliveries and deliveries at home conducted by trained health personnel) and receiving postnatal care by a skilled provider after delivery. As part of maternal health care services, ANC, delivery and PNC provided by a doctor, nurse, FWV or CSBA were considered as skilled providers. In this study ANC and PNC were defined as receiving the recommended minimum of 4 ANC visits and any PNC within 42 days of delivery from a skilled provider and skilled birth attendance during delivery as mentioned during the last pregnancy preceding the surveys. The household characteristics used to estimate the wealth index included source of water, electricity supply, sanitation facility, and main roof, wall and floor material. The asset variables included durable goods (such as television, radio, bicycle, and motorcycle), ownership of house, land and cattle [26].

#### Explanatory variables

The role of individual factors such as mother’s age, parity, socio-economic status, poverty index and husband’s level of education were examined [27]. Adjustments were made for the cluster design.

### Statistical analysis

All analyses were conducted using Stata 11. First, the descriptive statistics were calculated. The proportion of mothers who used each of the three maternal health services from skilled providers in each performing areas was presented. Chi-square test was used to test if the change from baseline to endline survey was significant. Descriptive analysis was conducted to understand the main source of skilled provider for the three maternal health services. Socio-economic status (SES) was determined using principle component analysis on the asset variables to calculate a household wealth status index [28,29]. This household wealth index was used as a proxy indicator for household wealth status in this analysis. Households were categorized from the poorest to the richest groups corresponding from the lowest to the highest quintiles. Individuals were sorted by the asset index of the household and placed into the respective quintile.

To investigate the effect of the intervention separately in three performing areas between two periods, i.e. baseline and end line, three separate logistic multivariate regression models for the three areas were analyzed. Tests of statistical significance and calculation of odds ratios and confidence intervals were performed using logistic multivariate regression. The covariates for the regression model were age of the mother, education of the mother and their husband, SES and parity. Clusters effect was also adjusted during the process of running the models. Logistic regression analysis was used to determine if the difference between the baseline and the endline variable estimate was statistically significant when adjusted for independent variables by the performing areas. For 4 + ANC from skilled providers the reference group was ‘ANC from unskilled providers’, for skilled attendant at delivery the reference group was ‘unskilled attendant at delivery’, and for PNC from skilled provider the reference group was ‘PNC from unskilled provider’. An effect of interaction was conducted to compare the changes in difference between baseline and endline survey period among the three performing areas for the three maternal health services. Test of statistical significance (< 0.05) and calculation of odds were conducted to assess the strength of the changes in all the performing areas after introducing the intervention. The odds between the endline and baseline surveys for each performing areas were further compared between ‘high and low’ and ‘medium and low’ performing areas.

### Ethical considerations

The study obtained the ethical approval from the Research Review Committee and Ethical Review Committee of icddr,b. Informed written consent was obtained from all study participants before participation. The informed consent included the purpose of the study, why the mother was selected, what will be asked, possible benefits and risks and their voluntary participation. All information was kept confidential.

## Results

### Background characteristics of mothers

A total of 894 and 900 mothers from the high performing areas, 1269 and 1500 mothers from medium performing areas, and 995 and 1031 mothers from the low performing areas were interviewed in the baseline and endline surveys respectively. The mothers were comparatively similar in terms of the major socio-economic indicators between the baseline and end line surveys across the performing areas (Table 1). Majority of the mothers were between 20–29 years of age, however more than 20% of the mothers were ≤ 19 years of age. Around half of the mothers from the low performing areas had no schooling during the baseline and end line period. More than 15% of women from the high and medium performing areas have had formal education to the level of ≥ 8^th^ grade whereas only 6% mothers from the low performing areas have formal education to ≥ 8^th^ grade. Of the 995 and 1031 mothers included in the baseline and endline surveys of the low performing areas, respectively, had the highest proportion of mothers (35%) in the poor quintile, whereas > 10% of the mothers from the high and medium performing areas were in the poor quintile. Interestingly, there was a significant difference among the performing areas for education and asset quintiles of the mothers during the baseline and the endline survey periods.

**Table 1:** **Socio demographic characteristics of 6-month delivered mothers of Shahjadpur sub-district during the baseline and the end line survey**

**Sociodemographic characteristics**	**Baseline (% of mothers)**			**End line (% of mothers)**		
	**High**	**Medium**	**Low**	**High**	**Medium**	**Low**
	**n = 894**	**n = 1269**	**n = 995**	**n = 900**	**n = 1500**	**n = 1031**
**Maternal age (in years)**						
≤19	22.2	23.0	22.7	21.1	20.3	21.4
20–29	64.2	62.6	61.1	61.1	60.9	59.9
≥30	13.7	14.4	16.2	17.8	18.8	18.6
*p-value*	0.540			0.931		
**Education level (in years)**						
No schooling	27.7	29.5	52.0	23.3	26.5	45.6
1–5	36.2	32.9	30.6	33.8	36.4	35.9
6–8	19.4	22.5	11.6	23.8	22.0	12.9
≥8	16.7	15.1	5.9	19.2	15.1	5.6
*p-value*	0.000			0.000		
**Occupation**						
Homemaker	94.7	96.5	92.4	92.0	92.1	91.1
Weaving	3.5	1.7	6.2	5.8	6.7	7.4
Others	1.8	1.8	1.4	2.2	1.2	1.6
*p-value*	0.000			0.227		
**Socio-economic status**						
Poor	11.0	14.4	35.3	10.2	14.9	35.4
Less Poor	16.1	20.4	23.2	14.4	20.5	23.8
Middle	23.0	20.7	16.2	18.6	21.1	19.0
Upper middle	21.8	22.7	15.0	24.1	21.9	14.1
Rich	28.1	21.8	10.4	32.7	21.5	7.8
*p-value*	0.000			0.000		

### Changes in maternal health care seeking behavior during baseline and end line surveys

Table 2 showed the changes in proportion of mothers who received skilled provider’s care of the recommended 4+ ANC, delivery and at least 1 PNC. Without adjusting for the independent variables, the cross tabulation (Table 2) indicated that mothers residing in all the three different performing areas used significantly more antenatal and postnatal care and received more skilled attendance at delivery from skilled providers at the end of the intervention. Regarding the use of antenatal and delivery care from skilled providers, the proportion of mothers of the high and medium performing areas was higher than the mothers of the low performing areas at the baseline. The levels of skilled provider care for 4+ ANC and delivery were expectedly high at the end of the intervention in these two performing areas while comparing with that of the low performing area. Nevertheless, these two indicators on use of maternal health services increased sharply from baseline to endline survey in low performing areas; 4+ ANC from skilled provider increased from 1.6% to 15.3% and SBA at delivery from 12.6% to 38.3%. The use of PNC from skilled provider was low in all the areas especially in low performing area (1%) at the baseline surveys. It increased to 50.1%, 39.5% and 19.8% in the high, medium and low performing areas, respectively, at the endline survey.

**Table 2:** **Maternal health care seeking behavior across the performing areas during the baseline and the end line survey**

**MNH care services**	**% of mothers**			
	**High**	**Medium**	**Low**	**Total**
	**% (n)**	**% (n)**	**% (n)**	**% (n)**
**ANC 4** ^**+**^ **by skilled providers**				
Baseline	13.1 (894)	7.3 (1269)	1.6 (995)	7.1 (3158)
End line	30.7 (900)	26.4 (1500)	15.3 (1031)	24.1 (3431)
*p-value*	0.003	0.000	0.000	0.000
**Delivery by skilled providers**				
Baseline	41.7 (894)	26.6 (1269)	12.6 (995)	26.4 (3158)
End line	54.8 (900)	44.6 (1500)	38.3 (1031)	45.3 (3431)
*p-value*	0.000	0.000	0.000	0.000
**PNC by skilled providers**				
Baseline	5.6 (894)	4.0 (1269)	1.0 (995)	3.5 (3158)
End line	50.1 (900)	39.5 (1500)	19.8 (1031)	36.3 (3431)
*p-value*	0.000	0.000	0.000	0.000

### Comparison of MNH care services across the performing areas

Tables 3, 4 and 5 presents multivariate results of use of skilled providers care during pregnancy, delivery and after delivery by selected independent variables. The covariates were adjusted for the three performing areas in the regression model.

**Table 3:** **Effect of the interventions on maternal health services among the performing areas for ANC 4+ and skilled providers**

**MNH services**	**High**		**Medium**		**Low**	
**ANC 4+ and skilled providers**	**Unadjusted OR**	**Adj. OR**	**Unadjusted OR**	**Adj. OR**	**Unadjusted OR**	**Adj. OR**
**Survey period**						
Baseline	1.0	1.0	1.0	1.0	1.0	1.0
End line	1.6 (1.2–2.2)	1.9 (1.1–3.3)	2.5 (1.8–3.5)	3.2 (1.9–5.3)	5.2 (3.0–9.2)	7.2 (3.6Z14.3)
**Maternal age (in years)**						
≤19	1.0	1.0	1.0	1.0	1.0	1.0
20–29	1.3 (0.9–1.9)	1.2 (0.7–2.1)	0.9 (0.6–1.2)	0.9 (0.6–1.2)	0.5 (0.4–0.8)	0.6 (0.4–1.0)
≥30	1.0 (0.6–1.6)	1.2 (0.6–2.2)	0.6 (0.4–0.9)	0.6 (0.3–1.0)	0.4 (0.2–0.9)	0.7 (0.3–1.6)
**Education level (in years)**						
No schooling	1.0	1.0	1.0	1.0	1.0	1.0
1–5	1.2 (0.8–1.9)	1.1 (0.7–1.8)	1.3 (0.9–1.9)	1.3 (0.8–2.1)	1.5 (1.0–2.4)	1.2 (0.6–2.1)
6–8	2.0 (1.2–3.1)	1.5 (0.9–2.4)	2 (1.3–3.0)	1.9 (1.2–3.0)	1.1 (0.6–2.0)	0.9 (0.4–1.9)
≥8	7 (4.2–11.6)	3.8 (2.0–7.1)	3.0 (2.0–4.6)	2.0 (1.1–3.5)	3.8 (2.1–6.9)	4.1 (1.2–13.7)
**Husband’s education level (in years)**						
No schooling	1.0	1.0	1.0	1.0	1.0	1.0
1–5	1.1 (0.7–1.7)	1.0 (0.7–1.5)	0.9 (0.6–1.3)	0.8 (0.6–1.1)	1.5 (1.0–2.4)	1.3 (0.8–2.3)
6–8	1.4 (0.9–2.3)	1.1 (0.6–2.1)	1.1 (0.7–1.7)	0.8 (0.5–1.3)	1.2 (0.7–2.3)	0.9 (0.4–1.9)
≥8	3.8 (2.6–5.7)	1.3 (0.8–2.3)	2.4 (1.7–3.4)	1.7 (1.0–3.0)	2.8 (1.7–4.6)	1.7 (0.6–4.7)
**Reproductive history**						
Primi-para	1.3 (.9–1.7)	1.3 (0.9–1.9)	1.5 (1.1–1.9)	1.1 (0.8–1.6)	2.4 (1.6–3.5)	2.0 (1.2–3.3)
Multi-para	1.0	1.0	1.0	1.0	1.0	1.0
**Socio-economic status**						
Poor	1.0	1.0	1.0	1.0	1.0	1.0
Less Poor	0.8 (0.4–1.7)	0.8 (0.4–1.5)	0.7 (0.4–1.2)	0.7 (0.4–1.3)	1.2 (0.7–2.1)	1.1 (0.6–1.9)
Middle	1.5 (0.8–2.7)	1.3 (0.6–2.7)	0.8 (0.5–1.3)	0.7 (0.4–1.2)	1.0 (.6–1.8)	0.8 (0.5–1.3)
Upper middle	1.4 (0.8–2.6)	1.0 (0.5–2.1)	0.9 (0.6–1.5)	0.7 (0.4–1.3)	1.0 (0.6–1.8)	0.8 (0.5–1.6)
Rich	4.2 (2.3–7.4)	2.1 (1.1–4.1)	1.5 (1.0–2.5)	0.9 (0.4–1.9)	1.4 (0.8–2.6)	0.7 (0.3–1.6)

**Table 4:** **Effect of the interventions on maternal health services among the performing areas for skilled delivery**

**MNH services skilled delivery**	**High**		**Medium**		**Low**	
	**Unadjusted OR**	**Adj. OR**	**Unadjusted OR**	**Adj. OR**	**Unadjusted OR**	**Adj. OR**
**Survey period**						
Baseline	1.0	1.0	1.0	1.0	1.0	1.0
End line	1.7 (1.4–2.0)	1.7 (1.3–2.3)	2.2 (1.9–2.6)	2.4 (1.8–3.2)	4.3 (3.5–5.4)	4.9 (3.3–7.2)
**Maternal age (in years)**						
≤19	1.0	1.0	1.0	1.0	1.0	1.0
20–29	1.1 (0.9–1.4)	1.3 (0.9–1.7)	1.1 (0.9–1.4)	1.4 (1.0–1.8)	0.8 (0.7–1.1)	1.0 (0.8–1.4)
≥30	0.8 (0.6–1.1)	1.2 (0.8–1.7)	0.9 (0.7–1.1)	1.2 (0.9–1.7)	0.7 (0.5–0.9)	1.1 (0.8–1.7)
**Education level (in years)**						
No schooling	1.0	1.0	1.0	1.0	1.0	1.0
1–5	1.2 (1.0–1.6)	1.1 (0.9–1.4)	1.3 (1.1–1.6)	1.1 (0.9–1.4)	1.5 (1.2–2.0)	1.3 (1.0–1.8)
6–8	1.7 (1.3–2.2)	1.2 (0.9–1.4)	1.7 (1.3–2.1)	1.3 (1.0–1.6)	3.1 (2.3–4.2)	2.7 (2.0–3.8)
≥8	4.5 (3.3–4.2)	2.6 (1.5–4.3)	4.1 (3.2–5.3)	2.7 (1.9–3.8)	4.8 (3.2–7.2)	3.8 (2.3–3.8)
**Husband’s education level (in years)**						
No schooling	1.0	1.0	1.0	1.0	1.0	1.0
1–5	1.0 (0.8–1.3)	0.9 (0.7–1.1)	1.3 (1.1–1.6)	1.1 (0.9–1.4)	1.1 (0.9–1.5)	0.9 (0.6–1.1)
6–8	1.3 (0.9–1.7)	1.0 (0.7–1.4)	1.6 (1.2–2.1)	1.2 (0.9–1.5)	1.2 (0.8–1.7)	0.6 (0.4–0.9)
≥8	2.7 (2.1–3.4)	1.2 (0.8–1.8)	2.3 (1.8–2.8)	1.1 (0.8–1.4)	2.7 (2.0–3.6)	1.0 (0.7–1.5)
**Reproductive history**						
Primi-para	1.7 (1.4–2.1)	1.7 (1.3–2.3)	1.5 (1.3–1.7)	1.4 (1.2–1.8)	1.8 (1.4–2.2)	1.7 (1.3–2.1)
Multi-para	1.0	1.0	1.0	1.0	1.0	1.0
**Socio-economic status**						
Poor	1.0	1.0	1.0	1.0	1.0	1.0
Less Poor	0.8 (0.5–1.1)	0.7 (0.5–1.0)	0.9 (0.7–1.2)	0.8 (0.6–1.1)	1.2 (0.9–1.6)	1.1 (0.8–1.5)
Middle	1.0 (0.7–1.4)	0.9 (0.7–1.2)	1.4 (1.1–1.9)	1.2 (0.9–1.7)	1.7 (1.3–2.3)	1.3 (0.9–1.9)
Upper middle	1.3 (0.9–1.8)	1.0 (0.7–1.4)	1.5 (1.1–2.0)	1.1 (0.8–1.6)	1.6 (1.1–2.1)	1.1 (0.8–1.6)
Rich	2.2 (1.6–3.1)	1.3 (0.9–1.8)	2.7 (2.1–3.6)	1.7 (1.1–2.5)	2.8 (2.1–3.6)	1.8 (1.1–2.9)

**Table 5:** **Effect of the interventions on maternal health services among the performing areas for PNC and skilled providers**

**MNH services**	**High**		**Medium**		**Low**	
**PNC and skilled providers**	**Unadjusted OR**	**Adj. OR**	**Unadjusted OR**	**Adj. OR**	**Unadjusted OR**	**Adj. OR**
**Survey period**						
Baseline	1.0	1.0	1.0	1.0	1.0	1.0
End line	12.3 (5.4–28.2)	23.6 (8.5–65.6)	8.8 (4.7–16.4)	8.5 (4.5–16.3)	2.2 (0.6–8.4)	3.6 (0.7–18.6)
**Maternal age (in years)**						
≤19	1.0	1.0	1.0	1.0	1.0	1.0
20–29	0.2 (0.02–1.3)	0.3 (0.04–2.9)	1.2 (0.6–2.3)	1.5 (0.6–3.5)	0.2 (0.1–1.1)	0.2 (0.03–1.1)
≥30	0.2 (0.02–1.5)	0.3 (0.02–1.3))	1.3 (0.5–3.1)	1.9 (0.6–6.2)	0.2 (0.04–1.0)	0.2 (0.03–1.8)
**Education level (in years)**						
No schooling	1.0	1.0	1.0	1.0	1.0	1.0
1–5	0.9 (0.3–2.3)	0.6 (0.2–2.2)	1.2 (0.6–2.6)	1.5 (0.7–3.6)	2.0 (0.8–5.0)	1.7 (0.5–5.1)
6–8	1.1 (0.3–3.5)	0.4 (0.1–2.0)	1.5 (0.6–3.5)	1.8 (0.6–5.0)	2.0 (0.7–5.8)	2.3 (0.5–10.3)
≥8	3.0 (0.7–12.2)	1.8 (0.3–12.6)	1.2 (0.5–2.7)	1.8 (0.6–5.5)	7.1 (0.9–55.2)	7.6 (0.6–96.3)
**Husband’s education level (in years)**						
No schooling	1.0	1.0	1.0	1.0	1.0	1.0
1–5	0.8 (0.3–2.2)	0.9 (0.3–3.1)	1.0 (0.5–2.2)	1.0 (0.4–2.3)	0.5 (0.2–1.1)	0.4 (0.2–1.0)
6–8	1.1 (0.3–4.2)	1.6 (0.3–8.1)	2.3 (0.6–8.0)	1.9 (0.5–7.5)	2.7 (0.3–21.5)	4.8 (0.4–53.0)
≥8	2.3 (0.7–6.9)	3.1 (0.6–16.1)	0.8 (0.4–1.6)	0.9 (0.3–2.4)	2.8 (0.8–10.0)	2.5 (0.4–15.2)
**Reproductive history**						
Primi-para	2.1 (0.8–5.4)	6.8)	1.2 (0.6–2.1)	1.6)	2.7 (1.1–6.8)	1.4 (0.4–4.6)
Multi–para	1.0	1.0	1.0	1.0	1.0	1.0
**Socio–economic status**						
Poor	1.0	1.0	1.0	1.0	1.0	1.0
Less Poor	0.2 (0.02–1.9)	0.2 (0.02–2.5)	2.0)	1.6)	1.8 (0.6–5.7)	4.4)
Middle	0.5 (0.1–4.5)	0.5 (0.04–5.4)	0.5 (0.1–1.8)	0.5 (0.1–1.9)	3.2 (0.8–12.1)	2.3 (0.5–9.9)
Upper middle	0.5 (0.1–4.7)	0.5 (0.04–3.7)	0.5 (0.1–1.7)	0.4 (0.1–1.6)	1.8 (0.6–5.5)	0.8 (0.2–2.9)
Rich	0.5 (0.1–4.2)	0.3 (0.03–3.7)	0.5 (0.1–1.8)	0.5 (0.1–1.9)	0.8 (0.3–2.4)	0.9)

After adjusting for the covariates in the model, the intervention was found to be positively affecting the receipt of 4 + ANC from the skilled providers compared with the mothers of the baseline surveys in all the three performing areas (Table 6). The results of the regression model showed that mothers of the low performing areas were 7 times (Adjusted OR, 7.20; 95% CI, 3.6–14.3) more likely to receive 4 + ANC from the skilled provider at the end of the intervention in comparison with the mothers who participated in the baseline survey. There was also a significant difference in the other two performing areas. The reporting on use of 4 + ANC from skilled providers by the pregnant mothers residing in the high and medium performing areas were 1.9 (Adjusted OR, 1.93; 95% CI, 1.1–3.3) and 3.1 times (Adjusted OR, 3.16; 95% CI, 1.9–5.3) respectively, higher from baseline to end line period.

**Table 6:** **Effect of the interventions on maternal health services among the performing areas**

**MNH services**	Adj. odds ratio*
**ANC 4+ and skilled providers by area**	
High performing area	1.93 (1.11–3.34)
Medium performing area	3.16 (1.87–5.32)
Low performing area	7.20 (3.63–14.29)
**Skilled delivery by area**	
High performing area	1.72 (1.27–2.33)
Medium performing area	2.40 (1.82–3.17)
Low performing area	4.85 (3.29–7.16)
**Skilled PNC by area**	
High performing area	23.64 (8.52–65.56)
Medium performing area	8.52 (4.46–16.29)
Low performing area	3.64 (0.71–18.62)

*Adjusted for maternal age, education, husband’s education, SES, reproductive history.

After adjusting for the covariates in the model, the intervention was found to be positively affecting the utilization of skilled attendance at child birth in all the performing areas (Table 6). The results of regression analysis showed that the mothers from high and medium performing areas were 1.7 (Adjusted OR, 1.72; 95% CI, 1.3–2.3) and 2.4 times (Adjusted OR, 2.40; 95% CI, 1.8–3.2) respectively, more likely to receive skilled attendance at delivery at the end of the intervention in comparison to that of the baseline survey.

Mothers from the low performing areas had 5 (Adjusted OR, 4.85; 95% CI, 3.3–7.2) times higher odds of receiving skilled attendance at delivery in the endline survey than the mothers of the baseline survey.

The overall use of postnatal care was found to be very low at the sub-district in the baseline survey. The results showed that the mothers from the high performing areas had higher likelihood of using postnatal care services than the mothers from the other two performing areas at the end of the intervention (Table 6). The odds of reporting the use of postnatal care among mothers of high and medium performing areas were about 23.6 and 8.5 times higher than the baseline whereas the odd ratio was 3.6 in the low performing area.

The comparative analysis between baseline and endline surveys, separately for all the performing areas, revealed that the intervention had an effect on the use of skilled care during ANC, delivery and PNC in all the areas. When this effect was compared for seeking 4 + ANC from skilled providers and use of SBAs at delivery between the ‘high and low’ performing areas, the changes were found to be significant (p < 0.0001). The increase of 4+ ANC from skilled providers and SBAs in the low performing areas after the integrated maternal health intervention was significantly higher than the increase in the high performing areas (4+ ANC from skilled providers: OR, 3.8; 95% CI, 1.9–7.6; SBAs: OR, 2.8; CI, 2.1–3.8). This confirmed that the change in the low performing areas was larger than the high performing areas from baseline to end line survey period.

### Source of skilled providers

**Figure 1:**
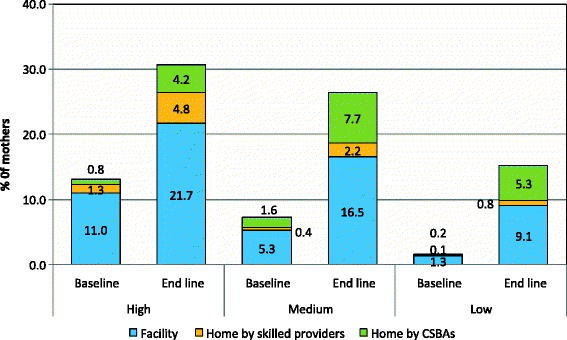
Source of skilled ANC by performing areas during the baseline and end line survey.

**Figure 2:**
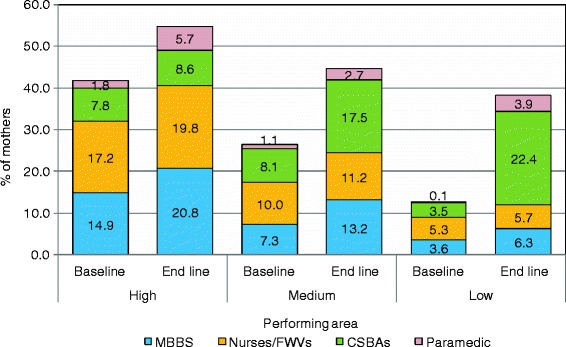
Source of skilled delivery by performing areas during the baseline and end line survey.

**Figure 3:**
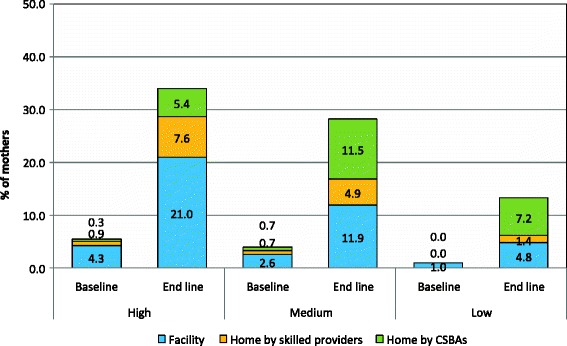
Source of skilled PNC by performing areas during the baseline and end line survey.

Figure 1 shows the proportion of mothers who received ANC from different skilled providers in three performing areas. Only 2% of the mothers each of the high and medium performing areas received their ANC from CSBAs at baseline and the proportion changed to nearly 10% during the endline survey. In the low performing areas the result was an increase from 0.3% to 6% from baseline to endline. Mothers living in the high performing area tended to have MBBS doctors and nurses with midwifery skills attending their delivery. Utilization of CSBAs was low in the high performing areas and there was almost no increase in this utilization at the endline. The utilization of CSBAs changed significantly from baseline to endline (8% to 17.5%) in the medium performing area. Substantial difference in the utilization of CSBAs was also found among the mothers of the low performing area between baseline and endline surveys (3.5% vs. 22.4%) at delivery (Figure 2). Figure 3 showed the proportion of mothers who received PNC from different skilled providers in three performing areas. Only 1% of mothers each of the high and medium performing areas received their PNC from CSBAs at baseline and the proportion changed to nearly 13% and 16% in the high and medium performing areas, respectively. In the low performing areas, it changed from 0% to 8.6% from baseline to endline.

## Discussion

The study findings revealed that the increase from baseline to endline in using CSBAs at ANC, delivery and PNC was significant across all the performing areas. The logistic multivariate regression models showed that there was a larger and stronger effect size between the baseline and endline period on maternal health skilled care in the low performing areas compared to the high performing areas. The baseline results revealed that the use of skilled providers care during pregnancy, delivery and after delivery was extremely low prior to the MNH intervention in the low performing areas. Only 1.6% of mothers received 4+ ANC from skilled provider, 12.6% delivery was conducted by SBAs and only 1% mothers received PNC from a skilled provider. In the endline survey 15.3% mothers sought 4+ ANC from skilled provider, 38.3% deliveries were conducted by SBAs and 19.8% sought PNC from skilled providers in the low performing areas. Prior to the intervention, the coverage of CSBAs revealed the shortage and also lack of availability of facility care in the low performing areas which presumably influenced the low use of skilled care by the mothers of low SES of these areas. The significant improvement in the use of skilled provider’s care could be partly attributed to the increased coverage of CSBAs from the intervention arm. The stratification of SES indicated that the highest proportion of poor mothers was in the low performing areas.

This study did not associate the improvement of skilled care with the mothers of low SES. Nevertheless, the availability of CSBAs coupled with counseling by CSBAs and CSGs could have contributed to the use of skilled providers care during pregnancy, delivery and after delivery by mothers low SES.

Some studies stated that insufficient human resources are one of the main factors for receiving skilled providers care in remote rural areas [30,17]. In areas of low coverage of facility care, a cadre of community health workers working in a public and private setting may be the most effective way to reach the underserved population [31]. This intervention is followed by appropriate selection of local women to be trained as CSBAs by NGO and government, use of the government curricula and setting for CSBA training, reorientation of the CSBAs, joint supervision and support for CSBA’s activities by government and NGO. Over the last decades, various strategies had been adopted to improve availability [32], accessibility [33] and affordability [34,35] of skilled delivery attendants. For example, Indonesia focused on home-based midwifery, while Cuba, Sri Lanka and Kerala improved a broader range of health services, including maternity care [32]. However, there is lack of investigation on which strategy works and how it works to reach the lower socioeconomic groups [36]. The reported increase in receiving skilled care from CSBAs during delivery after the intervention in low performing areas suggested the importance of extending healthcare to underserved populations at the community level. Although this study did not relate the use of skilled care to adverse pregnancy outcomes, other interventions found a reduction in mortality rates among women and children after increased training and mobilization of community health nurses [37,38].

Even when there is a broader range of health services, the geographic distribution of health care services acts as a major barrier to the access of skilled care by mothers [39]. Long distance to facilities with CEmOC, poor road infrastructure and transportation are the obstacles to receiving maternal health care from facilities especially in the remote areas of this study. In some of the low performing areas of this sub district, there are no roads to the CEmOC, the areas are surrounded by rivers or existing roads are not in use due to road quality or disruption due to the monsoon season. Some studies found that the distance to health facilities was a common reason for not seeking ANC from health facilities by the poor [40]. It is likely that mothers of these low performing areas would not be able to access the health care services. Lack of transport and restricted mobility due to pregnancy, means that women must give birth with trained attendants who are intended to have home deliveries. At the end of the intervention the mothers of the high performing area were less likely to use CSBAs compared with the mothers of the medium and low performing areas. There were significant effects on use of CSBAs at birth in medium and low performing areas. These findings revealed the significant role of the intervention in influencing skilled attendants at birth.

Lack of CSBAs was reported in the low performing areas at the baseline, and as expected the availability of health care facilities and skilled private practitioner services are mostly concentrated in high and medium performing areas. As a result, at the baseline mothers of the high and medium performing areas tended to report higher level skilled care compared with the mothers of the low performing areas. This was a potential reason for the higher proportion of mothers of these two performing areas to improve their maternal health skilled care compared to the mothers of the low performing areas at the endline. This is consistent with the statement that the availability of skilled services will influence the improvement in different areas differently [41]. This finding is particularly important for the collective coverage of both community and facility level skilled care targeting the remote areas. On the contrary, there was a larger and stronger effect size between the baseline and endline period on maternal health skilled care in the low performing areas compared to the high performing areas. In the logistic regression analysis, the increase of 4+ ANC from skilled providers and skilled birth attendants during delivery in the low performing areas due to the integrated maternal health intervention was significant relative to the increase in the high performing areas. This suggested that an integrated maternal health intervention with an adequate number of community skilled providers could contribute to serving the underserved population.

### Study limitations and strength

The study is focused on skilled providers care for maternal and neonatal health within the context of one sub district in Bangladesh. To make it generalizable to other sub districts of Bangladesh the results of the intervention need to be scaled up. Although there was a significant change in utilization of skilled providers care during pregnancy, delivery and after delivery, however there is still a need for more motivation since a large proportion of mothers did not report for skilled providers care in the low performing areas. One of the major limitations in comparing the differences between the groups, was that the results were attributed only to the intervention rather than comparing the effect of unmeasured factors including a control group.

One of the strengths of this study is the integrated approach of evidence based maternal health interventions in a single setting. Although this paper did not correlate the results on the use of skilled care with adverse pregnancy outcomes, it could be hypothesized that integrated interventions at community level may contribute to a reduction of adverse pregnancy outcomes. Additionally, recall bias would not be a limitation of the study because the mothers who delivered in the previous six months were included in the baseline and endline surveys. Another important strength of this study was to show the evidence of Public Private Partnership (PPP) in improving skilled maternal health care.

## Conclusion

The intervention was able to set up a public private health system by training a cadre of private CSBAs in addition to the existing government CSBAs. Prior to the intervention there was inadequate number of government CSBAs in the low performing areas. The increased number of CSBAs, in addition to the integrated approach of the intervention, showed improvements in utilization of skilled provider care by mothers. The placement of the required number of NGO CSBAs in areas which suffered from inadequate facility care could be associated with improved skilled care during pregnancy, delivery and after delivery.

### Recommendations

Nonetheless, the government of Bangladesh alone is not able to provide the adequate number of CSBAs throughout the sub districts of Bangladesh due to lack of resources. The policy makers and program implementers in Bangladesh and other countries with similar context could adapt the strategies especially the PPP of the present study. In addition, health facilities are located at a distance, and are not connected by proper roads or transport facilities in the low performing areas of this sub district. Therefore, the transport cost for receiving health services may often not be affordable for the poor mothers residing in these areas which in turn, influences the maternal health outcomes. The government of Bangladesh should patronize the establishment of primary health care facility nearby to these areas. Additionally the community should be encouraged to establish a transport system in their locality for the poor mothers.

The local government should be aware about the improvement of maternal health care services that were achieved through this research project. In order to sustain these positive effects, the performances of the CSBAs must be continued. In the event of withdrawal of icddr,b as an external agency, the sustainability would only be possible by implementation of a ‘systematic inquiry’ system in collaboration with the local government, heath care providers and community people, towards achieving an effective social change.

## Abbreviations

ANC: Ante natal care
CDK: Clean delivery kit
CSBAs: Community skilled birth attendants
CSG: Community Support Group
CEmOC: Comprehensive emergency obstetric care
FWV: Family Welfare Visitor
FRSs: Field Research Supervisors
FWC: Family Welfare Centres
HH: Household
IRB: Institutional Review Board
icddr,b: International Centre for Diarrheal Disease Research, Bangladesh
MMR: Maternal mortality ratio
MNH: Maternal and Neonatal Health
MDGs: Millennium development goals
NGOs: Non Government Organizations
MBBS: Bachelor of Medicine & Bachelor of Surgery
PPH: Post partum hemorrhage
PPP: Public private partnership
RDs: Rural dispensaries
SES: Socio-economic status
SBA: Skilled birth attendants
UHC: Upazila (sub-district) Health Complex
WHO: World Health Organization
